# Floating mechanism of a small liquid marble

**DOI:** 10.1038/srep21777

**Published:** 2016-02-23

**Authors:** Chin Hong Ooi, Chris Plackowski, Anh V. Nguyen, Raja K. Vadivelu, James A. St. John, Dzung Viet Dao, Nam-Trung Nguyen

**Affiliations:** 1 Queensland Micro- and Nanotechnology Centre, Griffith University, 170 Kessels Road, Nathan 4111, Queensland, Australia; 2 School of Chemical Engineering, University of Queensland, Brisbane, Queensland 4072, Australia; 3 Eskitis Institute for Drug Discovery, Griffith University, 170 Kessels Road, Nathan 4111, Queensland, Australia

## Abstract

Flotation of small solid objects and liquid droplets on water is critical to natural and industrial activities. This paper reports the floating mechanism of liquid marbles, or liquid droplets coated with hydrophobic microparticles. We used X-ray computed tomography (XCT) to acquire cross-sectional images of the floating liquid marble and interface between the different phases. We then analysed the shape of the liquid marble and the angles at the three-phase contact line (TPCL). We found that the small floating liquid marbles follow the mechanism governing the flotation of solid objects in terms of surface tension forces. However, the contact angles formed and deformation of the liquid marble resemble that of a sessile liquid droplet on a thin, elastic solid. For small liquid marbles, the contact angle varies with volume due to the deformability of the interface.

It is well known that a small object can float on a liquid medium that has a lower density. This phenomenon is critical to a number of both naturally occurring and man-made processes, from water-walking and meniscus-climbing of insects and spiders^1^,^2^,^3^ to separating gold, diamond and many other valuable minerals from rocks by froth flotation using air bubbles^4^,^5^. For solid objects, numerous studies have verified the modified Archimedes principle that includes the role of the surface tension of the liquid. The weight of the floating object is balanced by the buoyancy and the surface tension force. The surface tension force is present at the three-phase contact line (TPCL) where the three interfaces meet. This force acts along the tangent to the air-liquid surface at the three-phase contact line and provides an upward thrust that augments the buoyancy force^6^,^7^,^8^,^9^,^10^. At the TPCL, the contact angle is described by the Young equation which links the specific surface free energies (i.e., the surface free energies per unit surface area). It is noted that the specific surface free energy and surface tension do not coincide for the solid-gas and solid-liquid interfaces as they do for the fluid-fluid interfaces^11^. Therefore, the specific solid-liquid and solid-gas surface energies cannot be described as vectors and cannot be considered as forces. The Young equation does not describe the force balance at the TPCL as does the Neumann triangle for droplets floating at the air-water surface. Thermodynamically, no force balance can be written for the triple line on a solid particle and the Young equation must be derived from the grand thermodynamic potential^5^. In fact, in the case of floating solid particles, when taking the variation of the functional of the grand thermodynamic potential to determine its extremum, one must consider the dependence of the variations of the particle mass center and TPCL (known as the transversality condition), because the TPCL can move along the particle surface only. For floating droplets, the TPCL can move freely and the two variations are independent, leading to the two scalar equations of the Neumann triangle projected onto the two axes of the coordinate system. So, there is one degree of freedom for the motion of the mass center and TPCL of floating particles while the degree of freedom is equal to two for floating droplets.

Numerous studies have been focusing on systems that involve a solid straddling between two fluids^9^,^10^,^12^,^13^ (Fig. 1A), three fluids^14^,^15^,^16^,^17^,^18^ (Fig. 1B) and more recently two fluids and a deformable solid base^19^,^20^,^21^,^22^,^23^ (Fig. 1C). However, fundamental understanding is lacking for a system that involves a liquid marble (Fig. 2A), except our recent preliminary study on the deformation of a floating liquid marble droplet^24^. Liquid marble can be formed by coating a droplet with hydrophobic powder^25^. The hydrophobic coating is porous and prevents physical contact between the liquid inside the marble and an outside liquid or solid carrier. Thus, the liquid marble can be considered as a non-wetting droplet, Fig. 2A. The present paper investigates the system of a floating liquid marble and compares it with other systems depicted in Fig. 1(A–C). Specifically, this paper analyses the force balance equation and the contact angles at the TPCL and presents scaling relationships.

Determining the apparent contact angle of a sessile liquid marble on a solid substrate can be challenging. The most popular preparation method of liquid marbles is rolling a droplet on a powder bed which coats the droplet with layers of microparticles. These particles may aggregate and give the liquid marble an inhomogeneous surface. In contrast to a sessile liquid droplet, the lack of a clearly defined surface impairs accurate inspection of the edges and the apparent contact angles of a marble. For a floating liquid marble, the situation is even worse as the deformed liquid surface completely obscures the TPCL, making visible light not suitable as illumination source for imaging. We solve this problem by using an X-ray computed tomography (CT) imaging technique to obtain the side view of the floating marble, which was similarly conducted in a previous study of a liquid droplet on a soft solid substrate^21^. This technique not only reveals the TPCL, but also the “hidden” yet well-defined liquid marble edges, Fig. 2B.

## Materials and Method

We used loose polytetrafluoroethylene (PTFE) powder with a nominal diameter of 1 μm (Sigma-Aldrich®) to form the liquid marble. Distilled water was dispensed onto the PTFE powder bed and rolled around until the droplet was completely covered. A pipette (Thermo Scientific Finnpipette 4500, 0.5–10 μL) dispenses the accurate volume of the droplet (with an uncertainty of ±4.3%). The liquid marble was then rolled around on a clean surface to dislodge excess powder from its surface. Next, the liquid marble was gently placed in a clear polystyrene container filled with distilled water using a teaspoon and allowed to float. The container is filled almost to the brim so that the liquid marble can roll off the teaspoon gently onto the water surface without dropping. As the liquid marble is merely coated with a very thin layer of loose PTFE powder, simply dropping the marble onto the water surface could destroy it. Once the marble floated on the surface, water was drawn out from the container using a syringe to reduce the water level and to create a clearance at the top of the container for the lid. The container was sealed with its lid to minimise evaporation throughout the experiment. The container was carefully positioned on the sample stage of the X-ray CT equipment (VersaXRM-500 High-Resolution 3D X-ray Tomography Microscope System, ZEISS® Xradia, Pleasanton, CA, USA), Fig. 3A. The sample stage was adjusted until the water surface film was at its thinnest. This measure ensures that the camera tilt relative to the water level in the container was minimised. The X-ray source was set between 80 to 120 kV while the optical magnification was set to 0.4 or 4X depending on the marble size. Figure 3B depicts the schematic setup of our experiment.

After positioning the sample, the magnification and X-ray parameters were optimised and the profile image of the floating liquid marble was acquired. The recorded image was subsequently processed to fit its surface profile and to measure the contact angles at the TPCL using ImageJ (National Institutes of Health, USA). All images were exported using the lossless TIFF format with a resolution of 1007 × 1007 pixels. The image acquisition and processing procedures were repeated for marble volumes of 1, 2, 5, 10, 20 and 30 μL. Each liquid marble volume was measured three times. All critical properties are determined at room temperature and atmospheric pressure (25 °C and 1 atm). The density and surface tension of deionized (DI) water are taken as *ρ*_*w*_ = 1,000kg/m^3^ and *γ*_*aw*_ = 0.072N/m, respectively. PTFE has a density of 2,200 kg/m^3^. Due to the limitations of the liquid marble preparation and transfer methods, the PTFE packing density and coating thickness cannot be accurately controlled. The inhomogeneity of packing density may induce errors in the effective surface tension of the liquid marble.

Supplementary Information Figure S1 shows an image of the loose PTFE powder on the liquid marble surface. The image taken with an optical microscope indicates that the porous hydrophobic layer has a large range of particle clusters from a few to more than microns due to the aggregation of the loose particles. The curved and complex bottom surface of the floating marble is not accessible for our current imaging techniques. Figure S1 shows the bottom view of a sessile liquid marble on a glass slide to approximate the submerged layer of the floating liquid marble. The depicted image might not represent the actual coating layer of the floating liquid marble. We hypothesise that the coating thickness is different for the submerged and the unsubmerged portion of the marble. The unsubmerged layer is only exposed to air, so the loose particles could build up forming a thicker layer. In contrast, the submerged layer is sandwiched between the liquids of the marble and the bath making the particles to assemble into a thinner layer. The difference in thickness can be seen in the side view of the raw X-ray image in Supplementary Information Figure S2. The PTFE coating shows up as a fuzzy layer at the edge of the marble due to intensity differences. The free layer is several times thicker than the submerged layer. The difference in thickness of the coating layer could translate into a difference in packing density and consequently different surface tensions. Additionally, the interface between the marble and the liquid bath is heterogeneous, as it contains a mixture of air pockets and PTFE particles which gives rise to a large apparent contact angle^26^,^27^.

**Figure 2:**
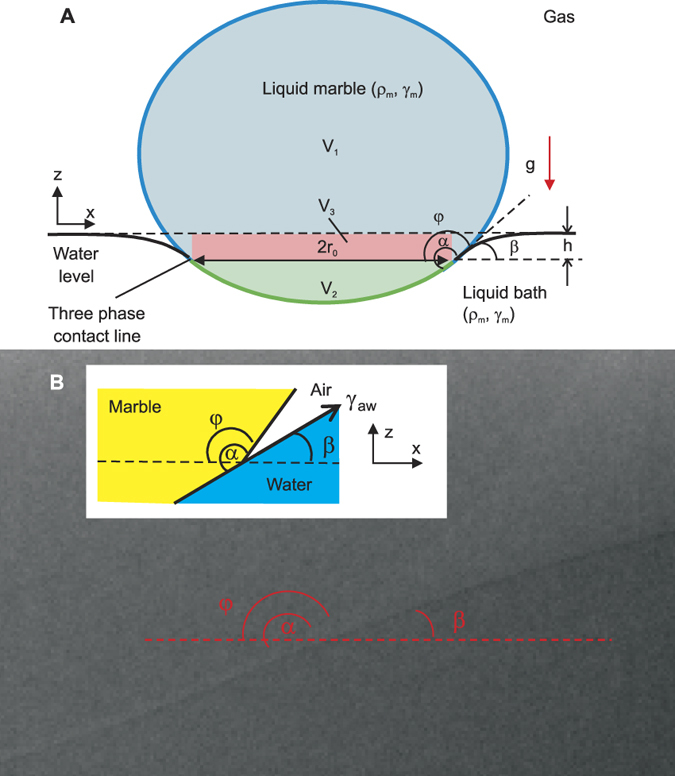
A liquid marble floating on a liquid bath: (**A**) Schematics of a floating liquid marble. Volumes are denoted as *V*_*1*_, *V*_*2*_ and *V*_*3*_. The blue and green lines bound *V*_*1*_ and *V*_*2*_ respectively. *r*_*0*_ is the contact radius of the liquid marble. *h* and *β* are the meniscus height and angle respectively. *α* is the liquid marble contact angle. *φ* is the liquid marble contact angle relative to the horizon. *γ*_*am*_ and *γ*_*aw*_ indicate the air-marble and air-water interfacial tensions respectively. *ρ*_*m*_ and *ρ*_*w*_ indicate the density of marble and water respectively. *g* indicates the direction of gravitational acceleration with a magnitude of 9.81 ms^−2^. (**B**) Magnified view of the TPCL X-ray image and its corresponding schematics.

**Figure 3:**
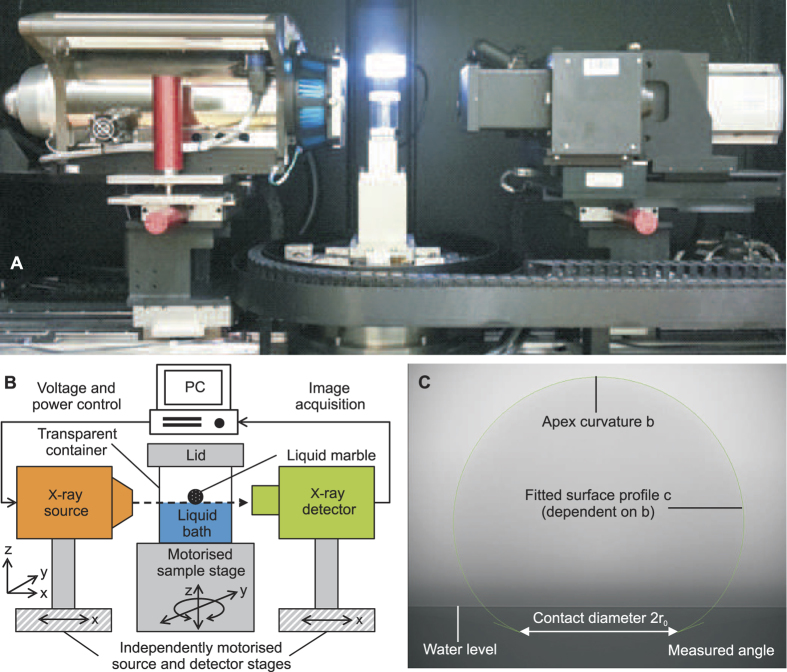
Imaging of a floating liquid marble: (**A**) The experimental setup with the X-ray source on the left, the X-ray detector with macro-lens and objectives on the right, and the 3D rotating stage in the middle. The liquid marble is placed in a clear polyethylene sample holder and placed on the sample stage between the X-ray source and the detector. (**B**) Schematics of the experimental setup. (**C**) Surface profile fitting of a floating liquid marble in a X-ray image. The “hidden” water surface is shown as a clearly defined edge. The gradual shading on the surface line is caused by the meniscus at the container wall. The apex curvature, *b* and inverse square of the capillary length, *c* are optimised such that the green line fits the marble image exactly. Low-bond axisymmetric drop shape analysis (LB-ADSA) then returns the measured angle and its base surface area, and consequently the contact radius, *r*_*0*_.

The effective surface tension of the marble *γ*_am_, the unsubmerged volume *V*_*1*_, the submerged volume *V*_*2*_ and the contact radius *r*_*0*_ (Fig. 2A) of the floating liquid marble were measured and calculated using the surface profile fitting procedure of LB-ADSA^28^, a freely available plugin for ImageJ. Manual fitting was required due to the inherently low contrast of the images and lack of a clear horizon at the three-phase contact line. Profile fitting starts at the apex of the marble. The apex curvature *b* was adjusted to fit the surface profile of the upper hemisphere. The height of the marble was then adjusted until the bottom end of the profile is level with the three-phase contact line. Next, the inverse square of the capillary length *c* = *ρ*_m_*g/γ*_am_, where *ρ*_m_
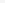
is the density of the marble and 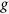 is the gravitational acceleration, was tuned to fit the lower hemisphere profile. Changing the capillary length *c* affects hemisphere profile, so *b* has to be lightly readjusted again. This iterative adjustment of *b* and *c* eventually yielded an accurate fitting of the marble surface profile. A set of values such as the contact radius *r*_*0*_, the inclination of the liquid marble relative to the horizon at the TPCL φ and unsubmerged volume *V*_*1*_ can be obtained upon the completion of the procedure. These results can be further verified by solving numerically the axisymmetric Young-Laplace equation. This popular form of solution involves parameterising the Young-Laplace equation into three first-order differential equations^29^,^30^,^31^. The required input parameters are the aforementioned apex curvature *b*, the inverse square of the capillary length *c* and the inclination φ.

## Theoretical Consideration

### Force balance

The total liquid marble volume *V* can be divided into the unsubmerged volume *V*_*1*_ and the submerged volume *V*_*2*_ (Fig. 2A), hence *V* = *V*_*1*_ *+* *V*_*2*_. For buoyancy force calculation, the cylindrical volume *V*_*3*_ is introduced. The buoyancy force produced by the liquid bath is equal to the weight of the volume of the displaced fluid *V*_*2*_ and the cylinder volume bounded between the surface of the contact line and the bath surface *V*_*3*_^7^,^9^. The cylinder has the radius of the three-phase contact line *r*_*0*_ and the same height *h* of the liquid bath meniscus. The volume *V*_*3*_ can be found by measuring both *h* and *r*_*0*_ from the acquired marble image: *V*_*3*_ = *πr*_*0*_^*2*^*h*, Fig. 2A.

In a macroscopic system, the condition of flotation is the balance between the weight *F*_w_ of the droplet and the buoyancy force *F*_b_. This force balance can be written in terms of volumes as 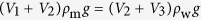 where *ρ*_m_ is the effective liquid marble density and *ρ*_w_ is the density of the carrier liquid^12^. In our experiment, the marble is filled with the same liquid as the bath. Neglecting contributions from the very thin hydrophobic coating layer, we assume that both liquids have the same density (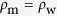). With some simplification, the force balance equation leads to *V*_1_ = *V*_3_, which is obviously not correct as the meniscus heights are usually very small, resulting in a much larger unsubmerged volume (*V*_1_ *>* *V*_3_), Fig. 2A. Therefore, a surface tension term must exist in the force balance of our case: 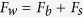, where *F*_*s*_ is the surface tension force. The existence of such surface tension force for an axisymmetric body had been rigorously proven by Keller^9^. In some previous works involving a three-fluid system^17^,^18^, this surface tension force was not taken into account. The setup was such that the meniscus angle is very small, thus producing a surface tension force which is much smaller than the buoyancy force.

Consequently, we hypothesise that the vertical component of the air-water surface tension along the TPCL yields a net upward force equal to *F*_s_. The liquid marble coating causes the liquid marble to behave like a deformable soft solid. The contact line dimensions of the present work are in the millimetre scale. At this scale, effects of line tensions (on the order of 10^−10^ N)^32^,^33^ are insignificant and therefore neglected. Also, the density of air relative to that of water is neglected in the subsequent analysis.

### Surface tension

At the TPCL, the air-water and marble-water interfaces are almost parallel. The air-marble interface branches out and inclines slightly more upward than the air-water interface. This configuration is similar to that of a liquid droplet resting on a thin elastic solid^34^ where an inflection point is present on the carrier solid. In our case, the inflection point must be at the TPCL as the liquid bath surface changes from a concave to a convex shape.

As the surface tension of the carrier liquid 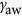 is known, its vertical component can be calculated from the known meniscus angle *β*. The force balance equation can be written as:


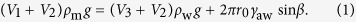


Assuming *ρ*_m_ = *ρ*_w_, *V*_2_ can be eliminated from both sides of the equation. Rewriting *V*_3_ in terms of *r*_0_ and *h* leads to a force balance equation with experimentally measurable quantities as:


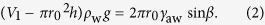


### Scaling analysis

We use the non-dimensional Bond number Bo as the independent variable for the scaling analysis. The Bond number represents the relative significance of the marble weight to the surface tension force:


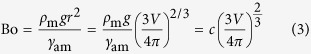


where *c* is the inverse square of the capillary length, a parameter used for liquid marble surface profile fitting as described in the materials and methods section, *r* is the radius of the non-deformed spherical liquid marble. Neglecting the weight the powder particle, the density of the marble 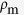
can be taken as the density of the marble liquid 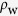. The Bond number scales with *r*^*2*^ and *V*^*2/3*^.

The constant contact angle α between a floating solid object and the liquid bath can be determined by straightforward measurements. With the known contact angle α, the meniscus angle β can be determined and a unique solution of the floating position can be found. However, our case of a floating liquid marble is different. The contact angle of a floating liquid marble *α* is not constant, because the marble is deformable.

We start off with the force balance equation: 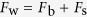. A small marble hardly depresses the water surface, yielding a small displaced liquid volume and a negligible buoyancy force. In our case, the surface tension force is about one or two orders of magnitude larger than the buoyancy force. Neglecting the buoyancy force leads to the force balance equation 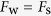.

The weight *F*_*w*_ scales with *r*^*3*^, while the surface tension force *F*_*s*_ scales with the contact radius *r*_0_ and sin *β*, therefore 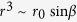. For a small liquid marble, β is small and the marble is almost spherical. Therefore the contact radius scales as 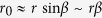. Substituting into the previous equation with further simplification yields 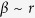. Bond number and *r* scale as 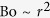, thus the scaling relationship between the meniscus angle and the Bond number is 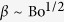.

The surface tension force *F*_s_ scales with *r*_*0*_sin *β*, therefore 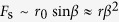. Since 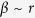 and 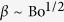, the scaling relationship between the surface tension force and the Bond number is 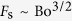. Normalising the contact radius *r*_*0*_ by the non-deformed marble radius leads to the dimensionless contact radius 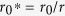. As elaborated above, the balance between the marble weight and the surface tension force leads to 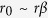, or in terms of dimensionless contact radius 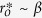 or 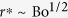. This scaling relationship was observed previously for of sessile marble on a solid surface^34^.

## Results and Discussion

**Figure 4:**
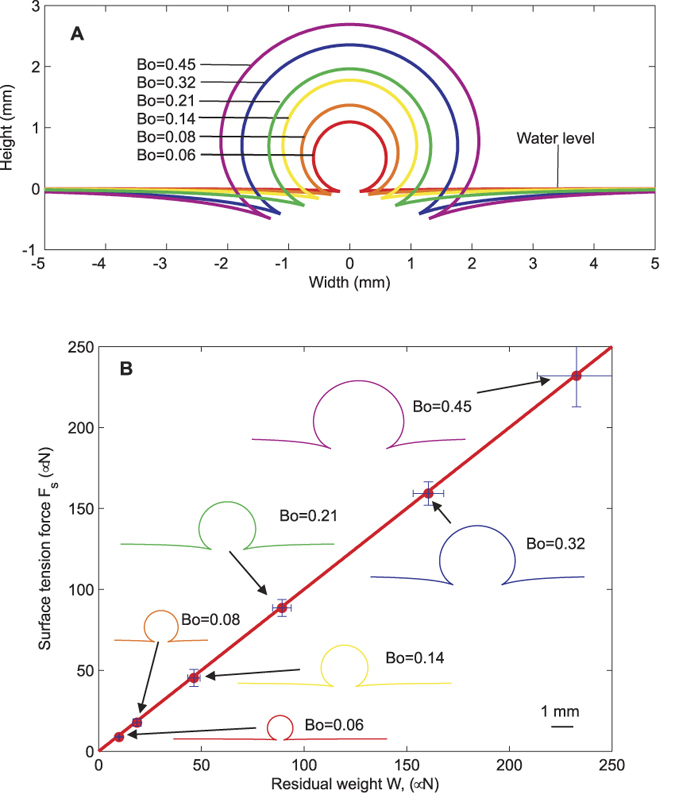
Measurement results: (**A**) Overlay plot of fitted marble surface profiles. The data labels indicate its corresponding Bond number as defined by Eq. (3). (**B**) Up-thrust arising from surface tension force 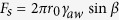 versus residual weight *W*_*r*_ = (*V*_1_ − *πr*_0_*h*)*ρ*_*w*_*g*. A straight line *F*_*s*_ = *W*_*r*_ is fitted onto this graph. Error bars indicate a 90% confidence level. The data point labels indicate the corresponding Bond number.

Figure 4A shows the measured profiles of floating marbles of various sizes. The measurement of the marble shape allows for the determination of the contact angle α and the meniscus angle β at the TPCL. The data in turns allows for the independent calculation of the upward thrust caused by surface tension *F*_*s*_ = 2*πr*_*0*_*γ*_aw_ sin *β* and the residual weight of the marble *W* = (*V* − *πr*_*0*_^*2*^*h*)*ρ*_*w*_*g*, where γ_aw_, ρ_w_ and 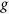 are the surface tension and density of water as well as the gravitational acceleration. The surface tension force *F*_*s*_, is the upward vertical force component that arises from the air-water interface tension. The residual weight *W* is the excess weight of the liquid marble not balanced by the buoyancy force alone. As depicted in Fig. 4B, the two force components determined independently through experimental data are found to have the same magnitude, cancelling each other, and enabling the marble to float on the liquid surface.

**Figure 5:**
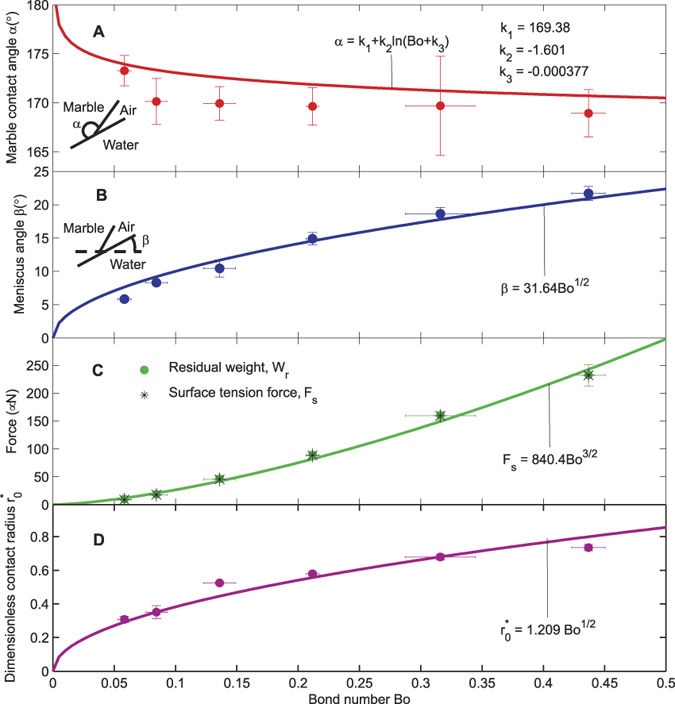
Measurement results: (**A**) Marble contact angle α versus Bond number Bo. (**B**) Meniscus angle β vesus Bond number Bo. The meniscus angle scales as *β *~ *BO*^1/2^. (**C**) Surface tension force and residual weight versus Bond number. The surface tension force scales as *F*_*s*_ ~ *Bo*^3/2^. (**D**) The dimensionless contact radius versus Bond number. The dimensionless contact radius scales as 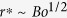. The error bars indicate a 90% confidence interval.

Figure 5A shows that *α* decreases with increasing Bond number. Unfortunately, the trend is not very clear due to the relatively large errors in the measurement. The shape of the liquid marble asymptotically approaches a perfect sphere with 0 contact radius as Bo approaches 0. Therefore the marble contact angle *α* approaches 180° with volume and Bond number approaching zero (Bo* *→* 0*). The marble contact angle *α* fits a logarithmic dependence in the form of 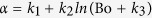 with a 180° intercept, where *k*_*1*_, *k*_*2*_ and *k*_*3*_ are curve fitting constants. Due to the limited amount of data, these constants only serve curve fitting purposes. More experimental data will be required for a functional analysis. The liquid marble contact angle *α* measured in our case is the apparent effective macroscopic angle. Microscopically, there is no physical contact between the marble liquid and the bath liquid^26^ because any contact will instantly result in the destruction of the marble. The liquid bath actually contacts a coating layer consisting of PTFE and air pockets. The liquid bath contact angle is not solely determined by the solid PTFE particles, because air pockets also contribute to the apparent contact angle^26^,^27^.

Figure 5B shows the experimentally measured meniscus angle *β* as function of Bond number. The data points of meniscus angle *β* fit a square root function of the Bond number Bo^1/2^ as predicted with the scaling analysis. Since there is no depression of the water surface with an asymptotic zero-volume marble, the meniscus angle *β* approaches 0° when the volume or the Bond number approach 0 (Bo → 0).

Figure 5C shows the two forces in the system as a function of the Bond number. The overlapping values of the two forces confirm the results depicted in Fig. 4B indicating that the surface tension force *F*_*s*_ is equal to the residual weight *W* of the marble regardless of its volume. As predicted from the scaling analysis, the surface tension force *F*_*s*_ scales with Bo^3/2^.

Figure 5D depicts the dimensionless contact radius versus the Bond number. The fitting curve of the experimental data follows the square root relationship predicted by the scaling analysis. It is interesting to note the that contact radius of a small floating liquid marble follows the same scaling relationship of a small liquid marble on a solid surface^29^,^34^. This scaling relationship is useful for describing the friction force of a moving and floating liquid marble in future work.

## Conclusions

We investigated the floating mechanism of liquid marbles using X-ray CT microscopy and theoretical analysis of the forces involved. We found that the flotation of small liquid marbles is predominantly affected by surface tension forces. The measurement of the apparent contact angles at the TPCL is not straightforward, due to the heterogeneous interface layer of the liquid marble that affects the effective surface tension. The configuration of the angles at the TPCL is similar to that of a sessile liquid droplet on a thin elastic solid whereby the carrier profile is smooth and continuous. The contact angle of a liquid marble varies with its volume due to the deformable interfaces. We hypothesise that marble contact angle decreases from the asymptotic value of 180 degree in the small Bond number range (Bo < 0.1) and remains almost constant with increasing Bond number. An improved preparation method for liquid marble would allow for future investigation in the sub-microlitre and millilitre scale and more conclusive results. Through scaling laws, we found that for a small, floating marble, the meniscus angle approximately scales with the square root of the Bond number 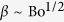
while the surface tension force scales with the Bond number raised to the power of 3/2 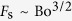. The contact radius also scales with the root of the Bond number as in the case of a liquid marble on a solid surface. In the microscopic scale at the TPCL, we hypothesise that the floating mechanism is the same as that of a solid straddling between two fluids due to non-wetting requirements for the existence of an intact floating liquid marble. Measurement at such a scale is beyond the scope of this paper but it would be a valuable addition to the understanding of this floating system.

## Additional Information

**How to cite this article**: Ooi, C. H. *et al.* Floating mechanism of a small liquid marble. *Sci. Rep.* **6**, 21777; doi: 10.1038/srep21777 (2016).

## Figures and Tables

**Figure 1:**
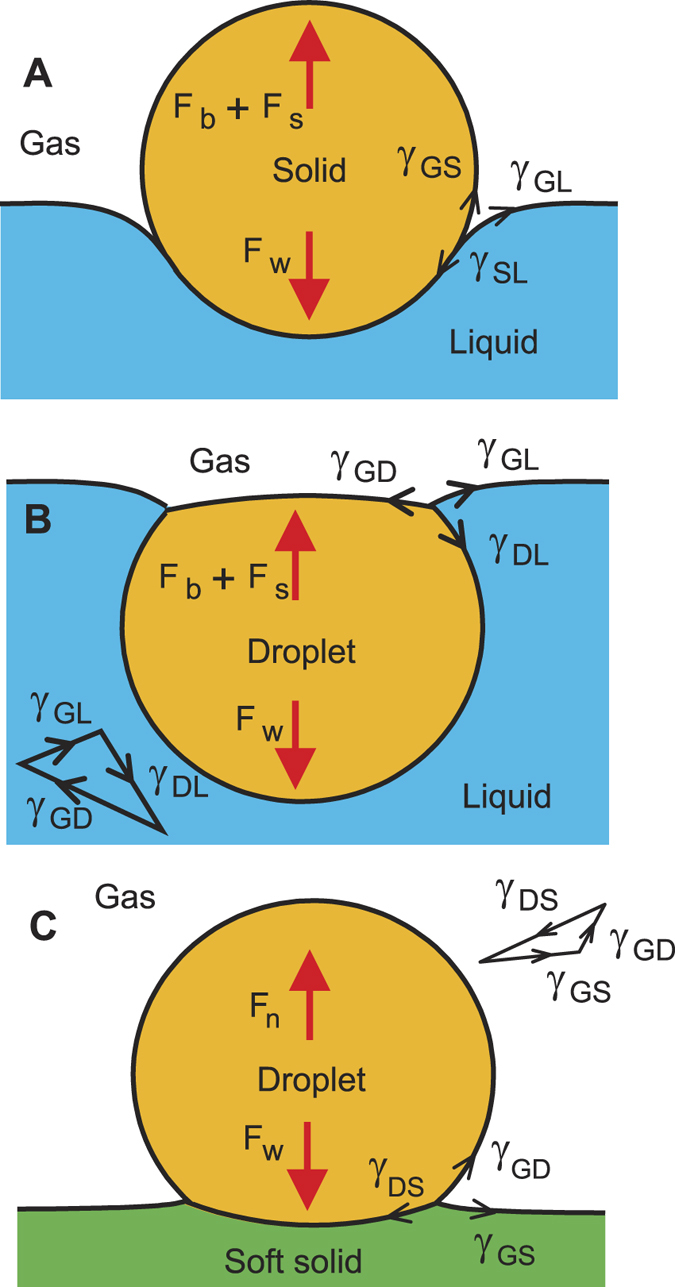
Different cases of floating objects. Key forces and interfacial tensions are shown. *F*_*w*_, *F*_*b*_, *F*_*s*_ and *F*_*n*_ denote gravitational, buoyancy, surface tension and normal forces respectively. γ_GD_, γ_GS_, γ_DS_, γ_GL_, γ_SL_ are gas-droplet, gas-solid, droplet-solid, gas-liquid and solid-liquid interfacial tensions respectively: (**A**) A non-wetting solid floats on a liquid bath. Young’s law is applied at the contact line, assuming a rigid floating object. (**B**) An immiscible, wetting liquid droplet floats in another liquid. Surface tension vectors form a Neumann triangle as shown. (**C**) A liquid droplet rests on a deformable soft solid^19^,^23^. Surface tension vectors form a Neumann triangle as shown.
